# MiR-29b/TET1/ZEB2 signaling axis regulates metastatic properties and epithelial-mesenchymal transition in breast cancer cells

**DOI:** 10.18632/oncotarget.22183

**Published:** 2017-10-31

**Authors:** Hua Wang, Xinglan An, Hao Yu, Sheng Zhang, Bo Tang, Xueming Zhang, Ziyi Li

**Affiliations:** ^1^ The First Bethune Hospital, Jilin University, Changchun, Jilin 130021, China; ^2^ College of Veterinary Medicine, Jilin University, Changchun, Jilin 130062, China; ^3^ College of Animal Science, Jilin University, Changchun, Jilin 130062, China

**Keywords:** MiR-29b, TET1, ZEB2, breast cancer, metastasis

## Abstract

MiR-29b has been reported to be both a suppressor and a promoter in breast cancer (BC) cells proliferation and metastasis. Significant efforts have been made to explain the seemingly contradictory effects of miR-29b on BC, but no answer has yet been clearly verified. In this study, we overexpressed and knocked down miR-29b in BC cell lines, modulated expression of its downstream target gene TET1 and downregulated a downstream target gene of TET1, ZEB2, to explore the regulatory mechanism of miR-29b in BC cell proliferation, migration and epithelial-mesenchymal transition (EMT). Our results showed lower expression of miR-29b in BC samples and cell lines. Functional assays showed that miR-29b overexpression resulted in a higher cell proliferation, greater colony formation, higher migration rate and EMT. A dual luciferase assay identified TET1 as a direct target of miR-29b. As the promoting effects of miR-29b in the proliferation and metastasis of MDA-MB-231 and MCF-7, knockdown of TET1 also led to increased proliferation, colony formation, invasion and EMT. Further, we found that TET1 bound to the promoter of ZEB2, and siTET1 enhanced ZEB2 expression. Disruption of ZEB2 expression inhibited BC cells proliferation, colony formation and invasion. Our results establish the miR-29b/TET1/ZEB2 pathway in BC cell proliferation, migration and provide a theoretical basis for further research on the molecular mechanisms and new clinical treatments for BC.

## INTRODUCTION

Breast cancer (BC) is one of the most commonly diagnosed cancers in women, with an estimated 1.2 million new cases worldwide each year, and it represents approximately 25% of cancers in women [1]. BC metastasis leads to most of the mortalities and has a critical role in the poor prognosis [2, 3]. The underlying molecular mechanisms in BC metastasis are still unclear. Hence, it is urgent to identify important molecules in cancer progression, which may be used to develop new diagnostic strategies and drugs.

During the past decade, microRNAs have been documented to be actively involved in various developmental and cellular processes, including organogenesis, differentiation and cancer [4–6]. MicroRNAs (miRNAs) are endogenous, small, non-coding RNAs, approximately 22 nucleotides in length [7]. They work as post-transcriptional regulators of gene expression by binding to the 3’-untranslated region (3’-UTR) of target mRNAs [8]. Recently, several studies have shown dysregulation of miR-29b in many types of tumours [5], such as gastric [9], breast [10] and prostate [11] cancer. As a member of the miR-29 family, miR-29b is generally recognized as a fundamental regulator of epithelial-mesenchymal transition (EMT), an event involved in cancer metastasis and chemoresistance. Previous studies showed that miR-29b modulates many target genes, such as the DNMT family [12, 13], oncogenes [14, 15] and tumour suppressor genes [16, 17]. Additionally, some DNA demethylases, such as the ten-eleven translocation (TET) family (TET1, TET2 and TET3) and thymine DNA glycosylase (TDG), are known to play important roles in biological phenomena and diseases that were previously poorly understood [18, 19]. Emerging evidence also suggests that the miR-29 family contributes to epigenetic regulation in cancer and primordial germ cell (PGC) development by targeting TET1, leading to global DNA hypermethylation [20, 21]. Since a preliminary bioinformatics analysis indicated the presence of multiple miR-29 binding sites on the 3’UTRs of TET1, we therefore sought to examine the regulatory role of miR-29 on demethylation pathways during epithelial-mesenchymal transition (EMT).

Additional evidence has illustrated that some transcription factors play important roles during EMT by binding to cis-regulatory elements in the promoter region of eukaryotic genes [22, 23]. It's been shown that zinc finger E-box-binding homeobox proteins, ZEB1 and ZEB2, two E-box-binding transcription factors, were involved in tumourigenesis of various malignancies [24, 25]. They can shift the epithelial phenotype of tumourigenic cells towards a more mesenchymal phenotype. ZEB1 and ZEB2 contain the helix-loop-helix motif allowing them to bind to the bipartite E-boxes within the E-cadherin promoter region with high specificity [26, 27]. As an upstream gene, ZEB2 reduces E-cadherin expression by binding to the E-cadherin promoter region to regulate EMT in BC cells [28, 29].

This study was conducted to investigate the role of miR-29b in BC cell growth, metastasis and EMT and explore the potential pathway of effects of miR-29b on BC behaviour in order to reveal molecular mechanisms of BC and provide a theoretical basis for clinical treatment of BC.

## RESULTS

### Expression of miR-29b in BC samples and cell lines

**Figure 1:**
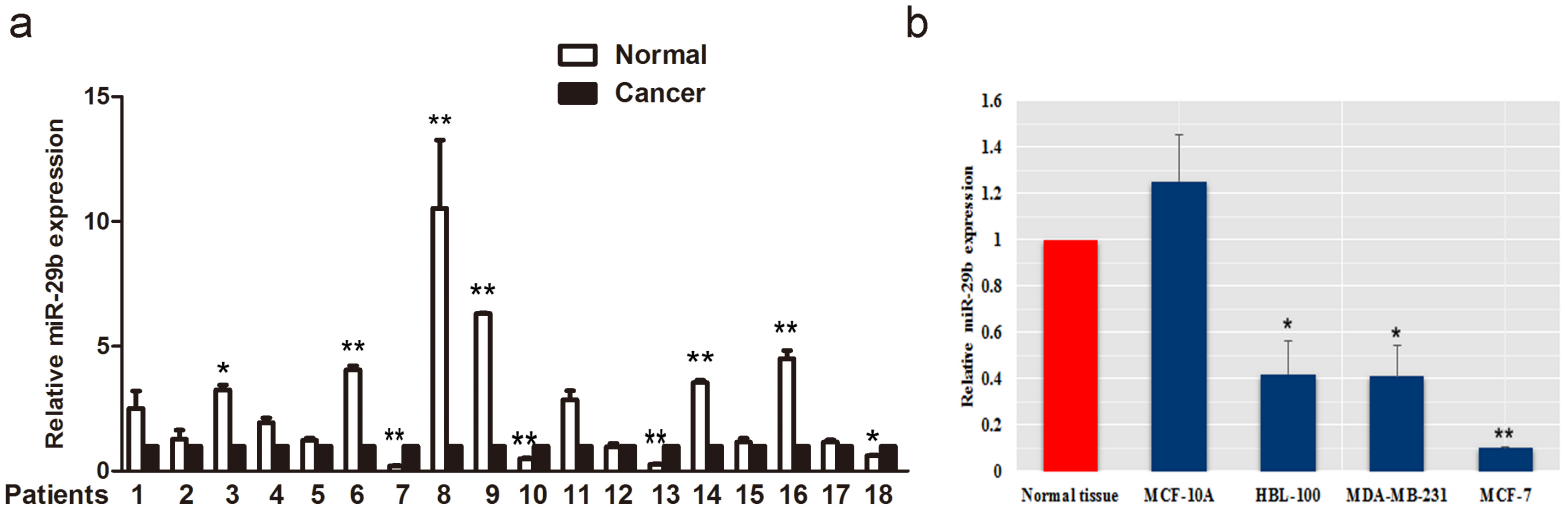
The expression of miR-29b in BC tissue and cell lines **(a)** The relative expression of miR-29b was lower in cancer samples than in adjacent normal tissue. **(b)** Levels of miR-29b expressed in BC cells relative to normal tissue. All data are expressed as the mean ± S.E.M. Asterisks denote significant effects; ^*^P < 0.05; ^**^P < 0.01.

We detected miR-29b expression in 18 BC samples and matched adjacent normal tissues using quantitative real-time PCR (qRT-PCR). In 18 samples, miR-29b expression were lower in 13 cancer samples. Among them, 6 cancer samples showed significantly lower miR-29b expression than adjacent normal tissues (*P* < 0.05, Figure 1a). Decreased miR-29b level were also observed in BC cell lines compared with that of the normal tissues (*P* < 0.05, Figure 1b).

### Exogenous miR-29b promoted BC cell proliferation and migration

**Figure 2:**
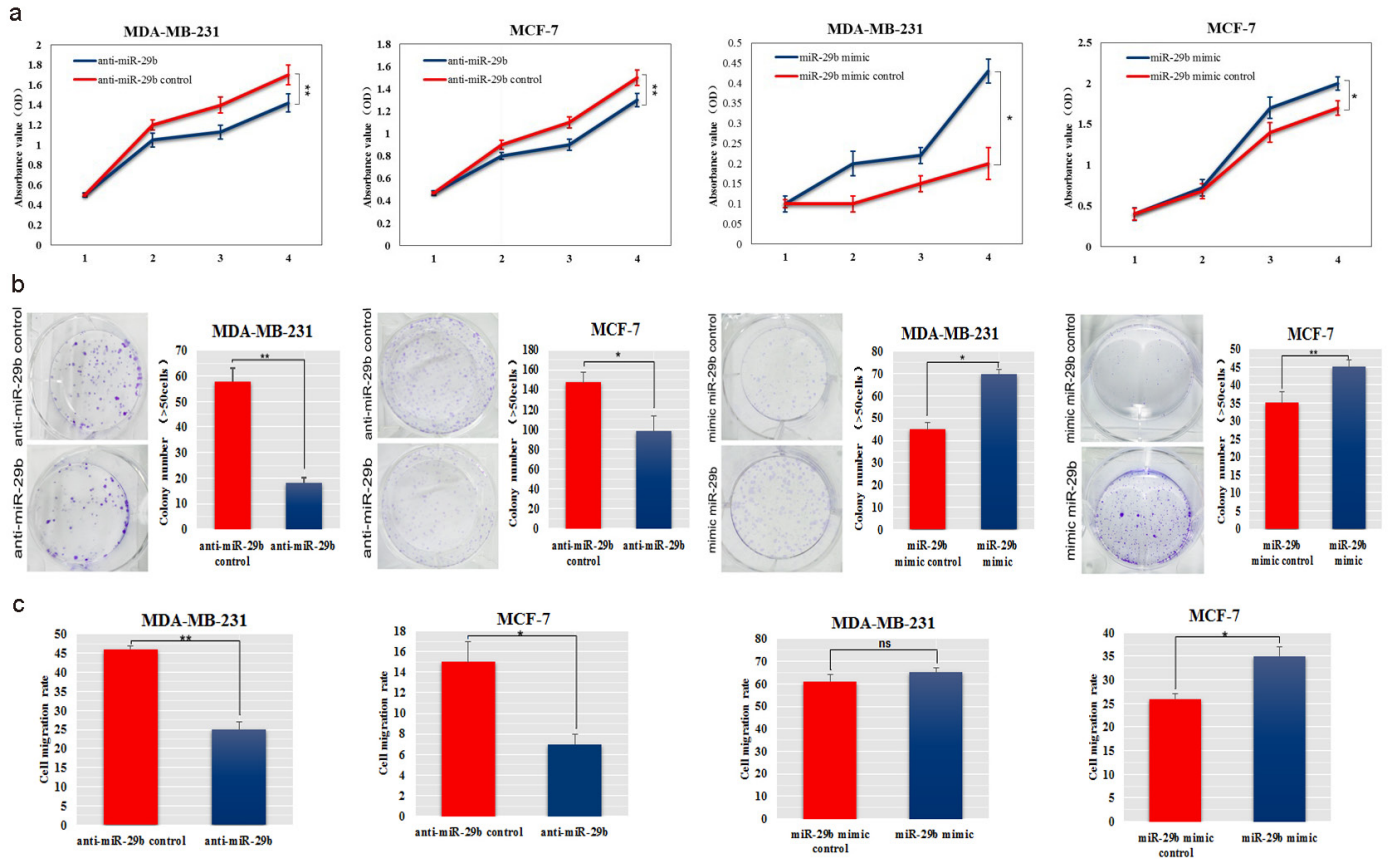
Ectopic expression of miR-29b promoted aggressive phenotypes in BC cells **(a)** The effect of miR-29b on cell proliferation was evaluated in miR-29b mimic or inhibitor-transfected MDA-MB-231 and MCF-7 cells. **(b)** Colony formation was detected after miR-29b transfection of MDA-MB-231 and MCF-7 cells. The numbers of colonies were scored in ten randomly selected fields. Each bar represents the mean of three independent experiments. **(c)** Cell migration rates in a wound healing assay were calculated in miR-29b mimic or inhibitor-transfected MDA-MB-231 and MCF-7 cells. All data are expressed as the mean ± S.E.M. Asterisks denote significant effects; ^*^P < 0.05, ^**^P < 0.01.

MiR-29b mimic was transfected into BC cell lines MDA-MB-231 and MCF-7 cells, and its effects on cellular behaviours and EMT-related gene expression were evaluated. QRT-PCR results showed that mimic transfection increased miR-29 expression significantly (Supplementary Figure 1a). We also found that miR-29b significantly decreased the expression of its target genes, C1QTNF6 and SPARC (Supplementary Figure 1b). CCK-8 and colony formation assays showed that miR-29b increased cell proliferation and significantly increased the colony formation ability in MDA-MB-231 and MCF-7 cells (*P* < 0.01 and *P* < 0.05, Figure 2a−2b). Invasion assays revealed significant induction of the migration of miR-29b mimic-transfected MDA-MB-231 and MCF-7 cells (*P* < 0.05 and *P* < 0.01, Figure 2c).

In contrast, the miRNA inhibitor anti-miR-29b was used to investigate the role of miR-29b depletion in MDA-MB-231 and MCF-7 cells. QRT-PCR results showed that miR-29b was decreased 3 to 4-fold after anti-miR-29b transfection, compared to control cells (Supplementary Figure 1b). After anti-miR-29b transfection, we detected an increase in C1QTNF6 (*P* < 0.05, Supplementary Figure 1b) and a rising trend in SPARC levels compared with those of the controls (Supplementary Figure 1b). Anti-miR-29b decreased the cell proliferation ability and markedly decreased colony formation in MDA-MB-231 and MCF-7 cells (*P* < 0.05 and *P* < 0.01, Figure 2a−2b). We also found a significant decrease in the migration rate of MDA-MB-231 and MCF-7 cells after transfection with the miR-29b inhibitor (*P* < 0.05, Figure 2c).

### MiR-29b regulated the expression of EMT related genes and 5hmc *in vitro*

**Figure 3:**
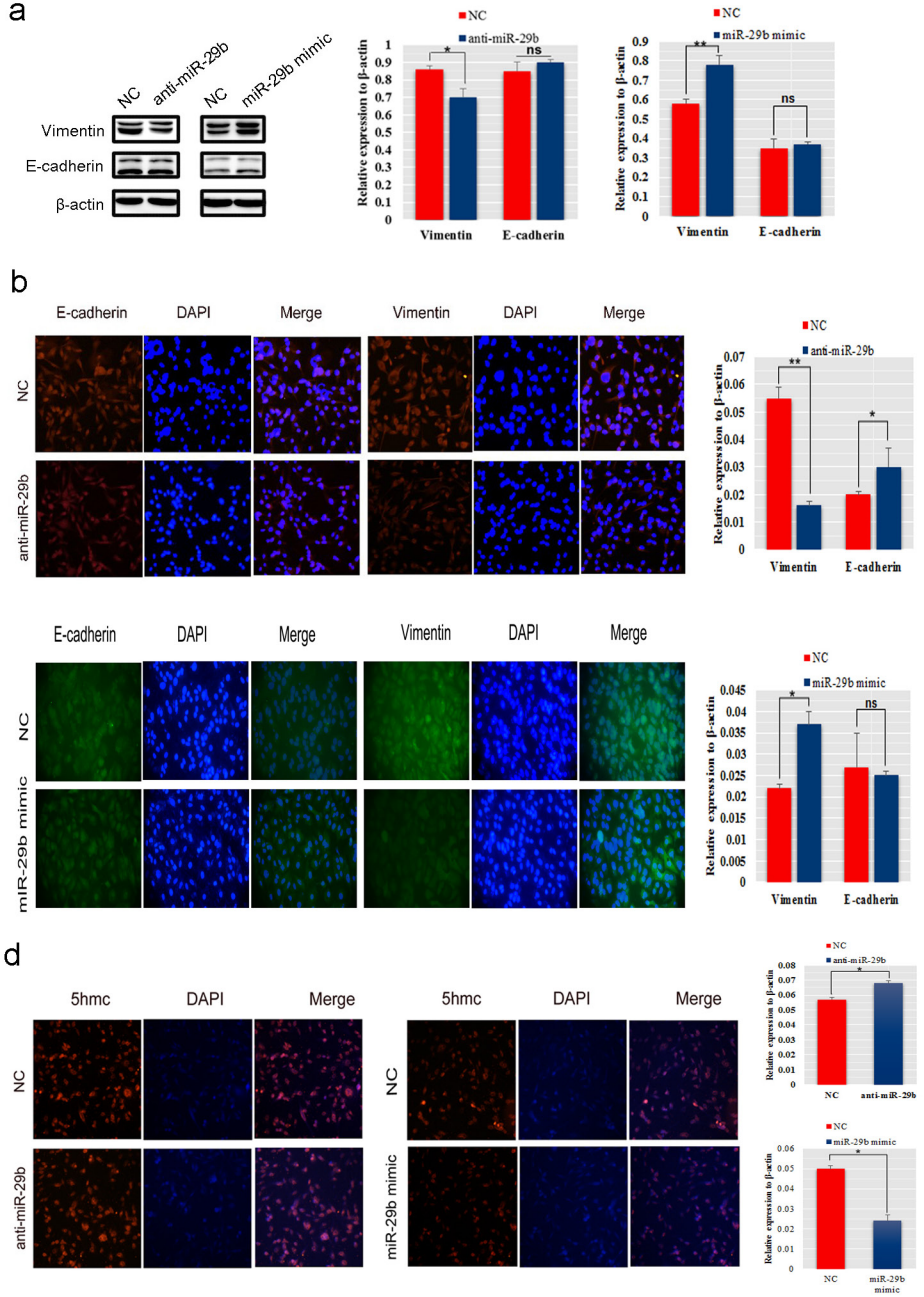
MiR-29b promoted EMT and regulated epigenetic changes in BC cells **(a)** Western blot analysis was performed to detect the expression of E-cadherin and Vimentin in MDA-MB-231 cells transfected with miR-29b inhibitor and MCF-7 cells transfected with miR-29b mimics. **(b**−**c)** An immunofluorescence assay was used to detect the expression level of E-cadherin and Vimentin in miR-29b inhibitor-transfected MDA-MB-231 cells or mimic-transfected MCF-7 cells. **(d)** The 5hmC level was detected in MDA-MB-231 cells transfected with miR-29b inhibitor and in MCF-7 cells transfected with miR-29b mimics. The immunofluorescence signal was quantified using densitometric scanning software, and the relative protein abundance was determined by normalization to the level of *β*-actin. All data are the means ± S.E.M; n = 3. ^*^P < 0.05; ^**^P < 0.01.

To understande the mechanism of miR-29b promoting of BC cells metastasis, we evaluated the genetic and epigenetic change in miR-29b mimic transfected MDA-MB-231 and inhibitor transfected MCF-7 cells. Western blot analysis showed that exogenous miR-29b overexpression resulted in an increase in the mesenchymal marker Vimentin (*P* < 0.01), while the miR-29b inhibitor induced a decrease in Vimentin (*P* < 0.05, Figure 3a). Interestingly, there was no obvious change in expression of the epithelial marker E-cadherin, both in miR-29b mimic- and anti-miR-29b transfections. Immunofluorescence assays of the anti-miR-29b transfection indicated that Vimentin was decreased dramatically (*P* < 0.01), while E-cadherin increased (*P* < 0.05, Figure 3b). Immunofluorescence analysis of the miR-29b mimic-transfection showed that Vimentin was significantly elevated (*P* < 0.05), while no significant difference in E-cadherin was observed (Figure 3c). Epigenetically, 5-hydroxymethylcytosine (5hmC) levels analysis results showed that the 5hmc level was much higher in miR-29b inhibitor-transfected MDA-MB-231 cells than in control cells and lower in miR-29b mimic-transfected MCF-7 cells than in control cells give another complementary proof to their interaction (*P <* 0.05, Figure 3d).

### MiR-29b directly targeted TET1

**Figure 4:**
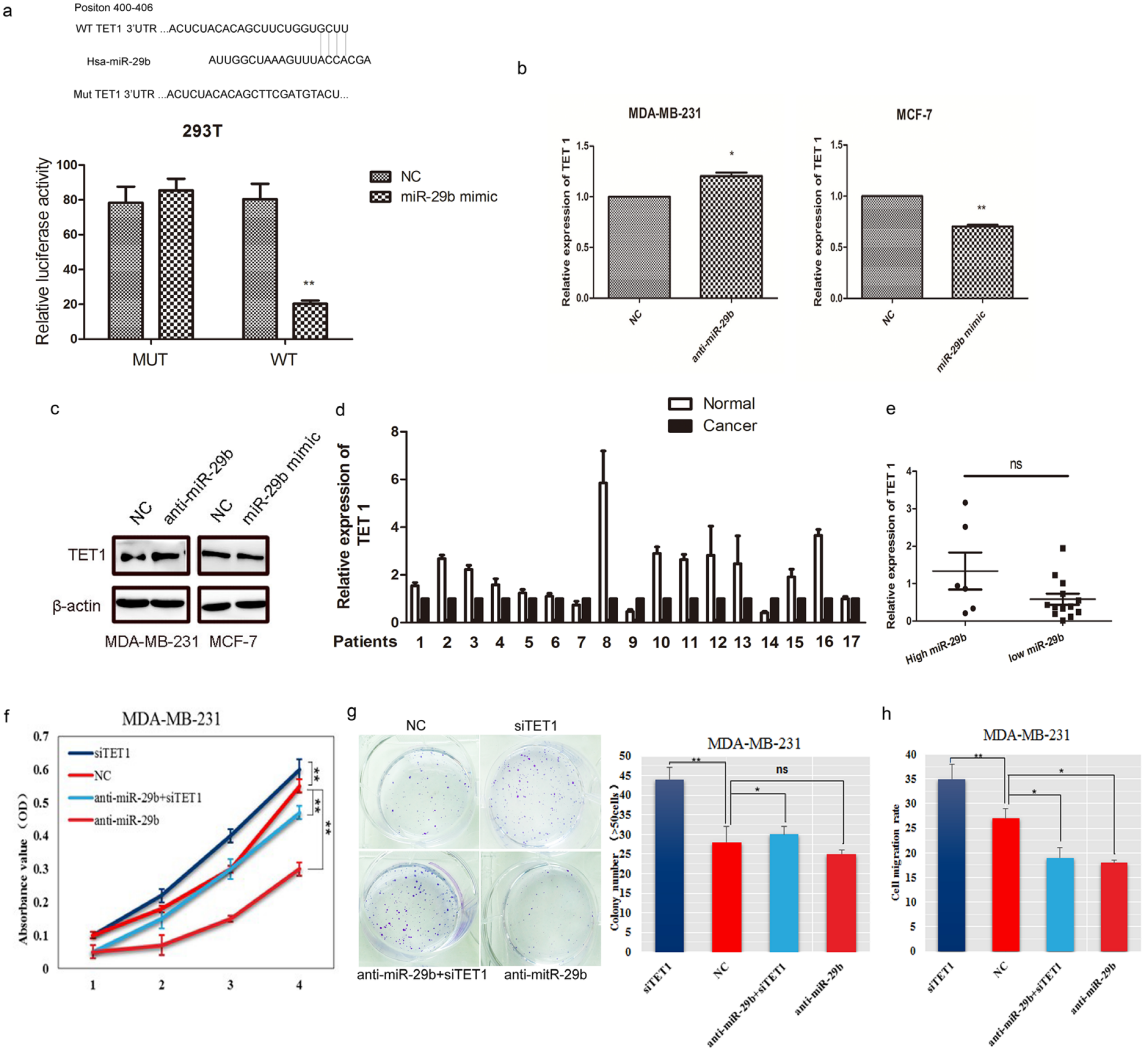
TET1 was a direct target of miR-29b **(a)** Structure of the human TET1 3’UTR containing wild−type and mutant miR-29b binding sites. Luciferase activity was downregulated in miR-29b mimic and wt TET1 3’UTR vector cotransfected 293T cells compared with miR-29b mimic and mt TET1 3’UTR cotransfected cells. **(b)** TET1 mRNA was decreased or upregulated in miR-29b mimic or inhibitor transfected MDA-MB-231 and MCF-7 cells. **(c)** Western blot analysis was performed to detect the expression of TET1 in MDA-MB-231 cells transfected with miR-29b inhibitor and MCF-7 cells transfected with miR-29b mimics. **(d)** TET1 expression in BC samples and adjacent normal tissues. **(e)** TET1 expression in miR-29b high- and low- expressing BC samples. The difference in TET1 expression was not significant (*P*=0.46). The high miR-29b expression level was above and the low miR-29b expression level was below the mean expression value of all of the samples. The horizontal line in the graph represents the mean of each group. **(f)** The CCK-8 assay was performed to quantify the proliferation rate in MDA-MB-231 cells transfected with siTET1, miR-29b inhibitor respectively, and cotransfected with both. **(g)** Colony formation was detected in cells transfected with siTET1, an miR-29b inhibitor cotransfected with both. The number of colonies were scored in ten randomly selected fields. Each bar represents the mean of three independent experiments. **(h)** Wound healing rates were numerized in cotransfected cells. Data are expressed as the means ± S.E.M; n = 3. ^*^P < 0.05; ^**^P < 0.01.

Using a bioinformatic analysis (based on TargetScan Human 6.2, PicTar and miRanda), TET1, which was previously identified as a BC metastasis-related gene, was predicted to be a potential target of miR-29b. A luciferase reporter assay was used to determine whether miR-29b can directly target the 3’UTR region of TET1. The wild-type target sequence (wt 3’UTR) and a mutated sequence (mt 3’UTR) were constructed in a luciferase reporter vector and then transfected into 293T cells with the miR-29b mimic. Luciferase activity was significantly decreased in wt vectors and miR-29b mimic cotransfecting 293T cells. A mutation in the putative miR-29b binding site in the TET1 3’UTR region abrogated this repression (Figure 4a), suggesting a direct interaction between miR-29b and TET1. The interaction was supported by our observation of the ability of miR-29b to suppress TET1 expression both at the mRNA and protein levels in MDA-MB-231 and MCF-7 cells (Figure 4b−4c).

To further explore whether miR-29b targets TET1, we detected TET1 expression in BC samples and in miR-29b high- or low- expressing group. The results showed that TET1 expression was lower in cancer samples than in adjacent normal tissues and that there was no significant difference in TET1 expression between miR-29b high- and low- expressing groups (Figure 4d−4e). Biologically, we then simultaneously co-transfected MDA-MB-231 cells with a miR-29b inhibitor and siTET1. We found that cell growth was decreased when the cells were transfected with the miR-29b inhibitor, but siTET1 could rescue the inhibited cell growth in MDA-MB-231 cells treated with both anti-miR-29b and siTET1 (*P* < 0.05, Figure 4f). The colony number of miR-29b inhibitor-transfected cells increased when cells were co-transfected with the miR-29b inhibitor and siTET1 (*P* < 0.05, Figure 4g). Our data also revealed that siTET1 could markedly rescue the migration rate of MDA-MB-231 cells (*P* < 0.01, Figure 4h). These data collectively indicated that TET1 is a target gene of miR-29b.

### SiTET1 promoted BC cell growth and migration

**Figure 5:**
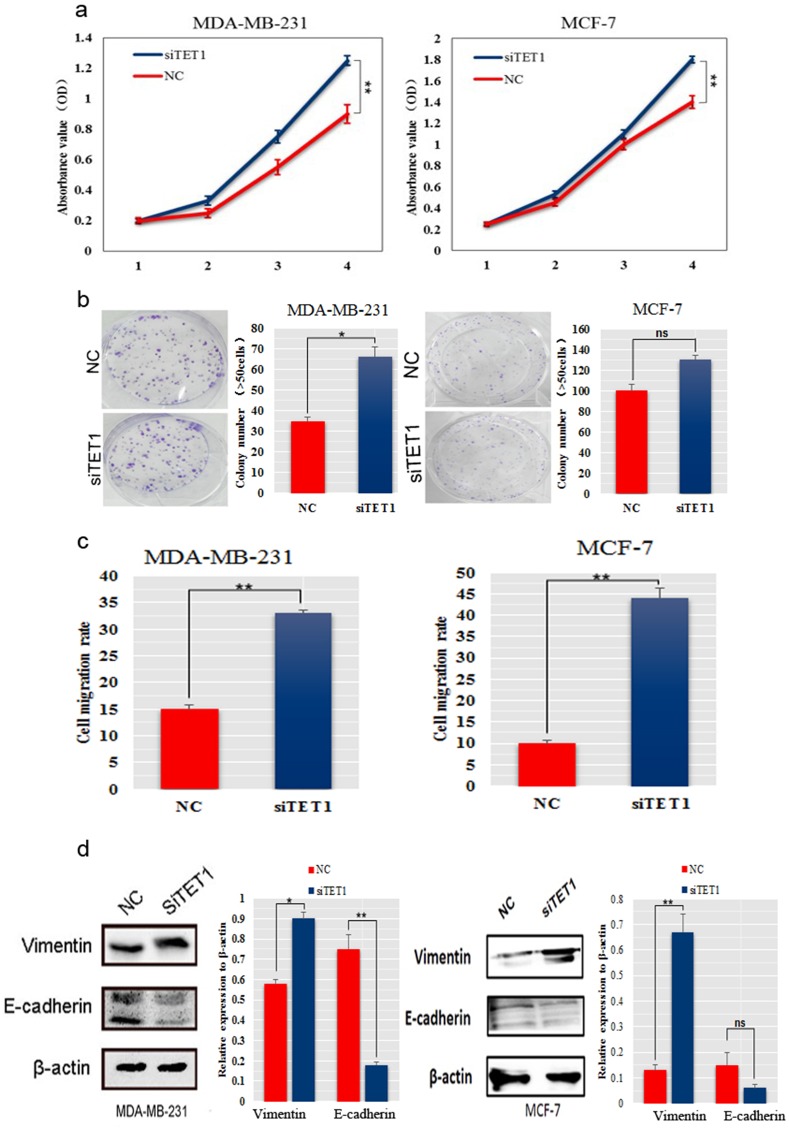
SiTET1 promotes the motility and invasion of BC cells **(a)** The effect of downregulating TET1 on cell proliferation was determined using the CCK-8 assay. **(b)** Colony formation assays for MDA-MB-231 and MCF-7 cells transfected with siTET1. The number of colonies were scored in ten randomly selected fields. Each bar represents the mean of three independent experiments. **(c)** Data from the wound healing assay for MDA-MB-231 and MCF-7 cells transfected with siTET1. Bars represent the migration rate of invading cells after transfection with siTET1. **(d)** Western blot was performed to detect E-cadherin and Vimentin expression in MDA-MB-231 and MCF-7 cells transfected with siTET1. Data are expressed as the means ± S.E.M; n = 3. ^*^P < 0.05; ^**^P < 0.01.

The observation that TET1 was a target gene of miR-29b (Figure 4a) led us to further investigate the roles of TET1 in BC cell proliferation and migration. We silenced TET1 in MDA-MB-231 cells using small interfering RNAs. The CCK-8 assay and colony formation analysis showed that knockdown of TET1 significantly increased cell proliferation and the colony formation ability, respectively (*P* < 0.05, Figure 5a−5b). An invasion assay showed an increase in the migration rates in the cells with knockdown of TET1 compared with control cells (*P* < 0.01, Figure 5c). Because we observed that miR-29b exerted a slight influence on EMT-related genes (Figure 3a), we examined whether its target gene, TET1, had a more pronounced effects on EMT-related genes. Notably, western blot analysis in MDA-MB-231 and MCF-7 cells showed that Vimentin was dramatically increased; while E-cadherin was considerably decreased in siTET1 transfectants (Figure 5d), suggesting that siTET1 could promote EMT.

### SiTET1 promoted EMT by binding to ZEB2

**Figure 6:**
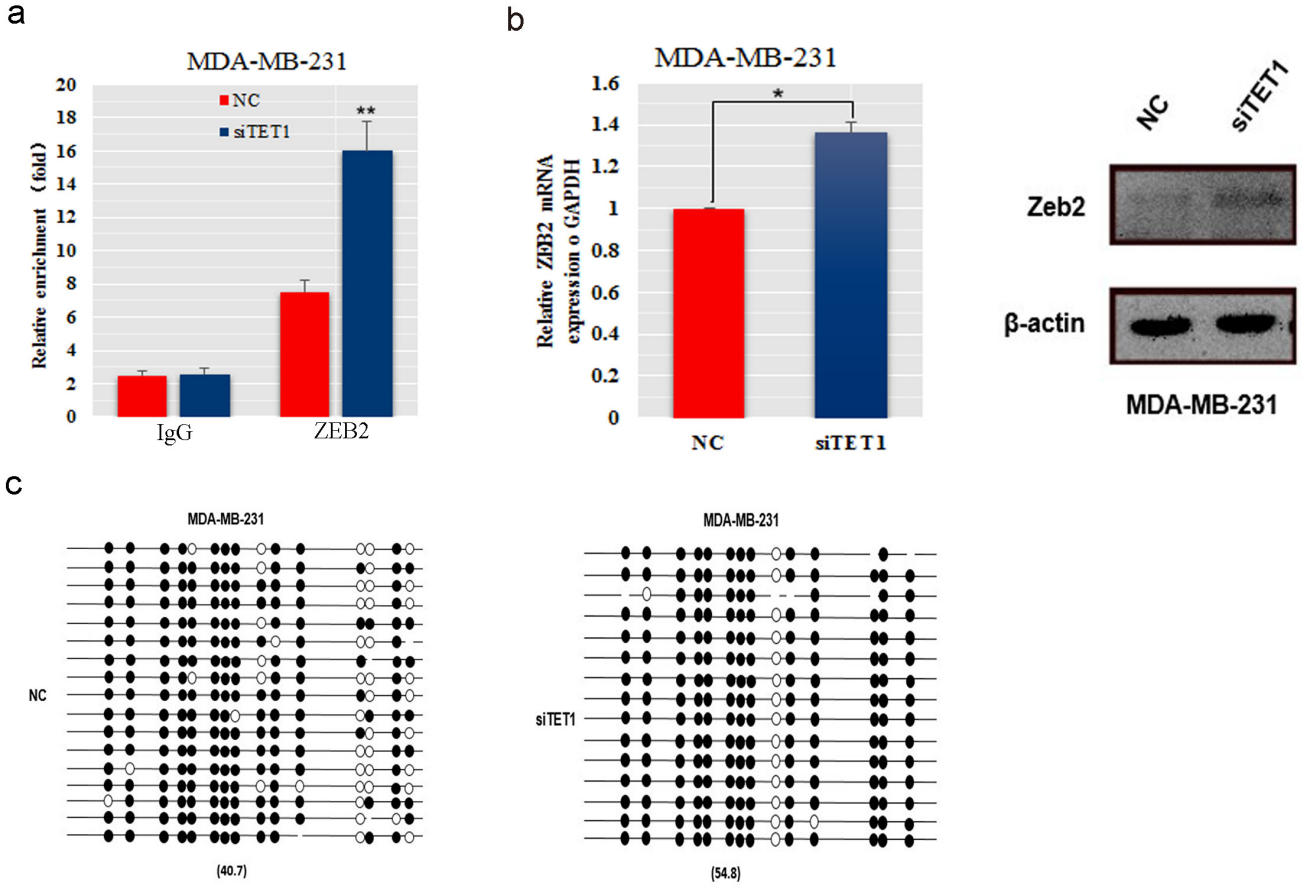
The expression and methylation levels of the ZEB2 gene in MDA-MB-231 cells transfected with siTET1 **(a)** Chip-qPCR was performed to detect TET1 protein binding to the ZEB2 promoter. **(b)** ZEB2 mRNA and protein was detected in MDA-MB-231 cells transfected with siRNA against TET1. The immunofluorescence signal was quantified using densitometric scanning software and the relative protein abundance was determined and normalized to the level of *β*-actin. **(c)** The ZEB2 gene methylation levels of TET1 siRNA-transfected MDA-MB-231 cells. Data are expressed as the means ± S.E.M; n = 3. ^*^P < 0.05; ^**^P < 0.01.

Changes in expression of EMT marker genes are caused by regulatory factors, including TWIST, SNAIL and ZEB. Previously CHIP-seq analysis of TET1 in mouse embryo showed that ZEB2 might be a downstream target gene of TET1 because its promoter region has numerous TET1 binding sites. To determine whether ZEB2 is a downstream target gene of TET1, we assessed the ability of TET1 to bind to the ZEB2 promoter in MDA-MB-231 cells. TET1 binding to the ZEB2 promoter was significantly increased when TET1 was knocked down (Figure 6a). Consistent with the markedly elevated level of TET1 binding in ZEB2 promoter region, we also found that ZEB2 mRNA and protein levels were decreased in MDA-MB-231 cells transfected with siTET1 (Figure 6b), which suggests that ZEB2 is a downstream target gene of TET1. Epigenetically, we detected that knockdown of TET1 led to an increase in the methylation level of the ZEB2 promoter (54.8%) compared with control cells (40.7%, Figure 6c). These results suggest that high binding of TET1 in the ZEB2 promoter region and high methylation downregulate ZEB2 transcription and translation expression.

### SiZEB2 inhibited BC cell growth and migration

**Figure 7:**
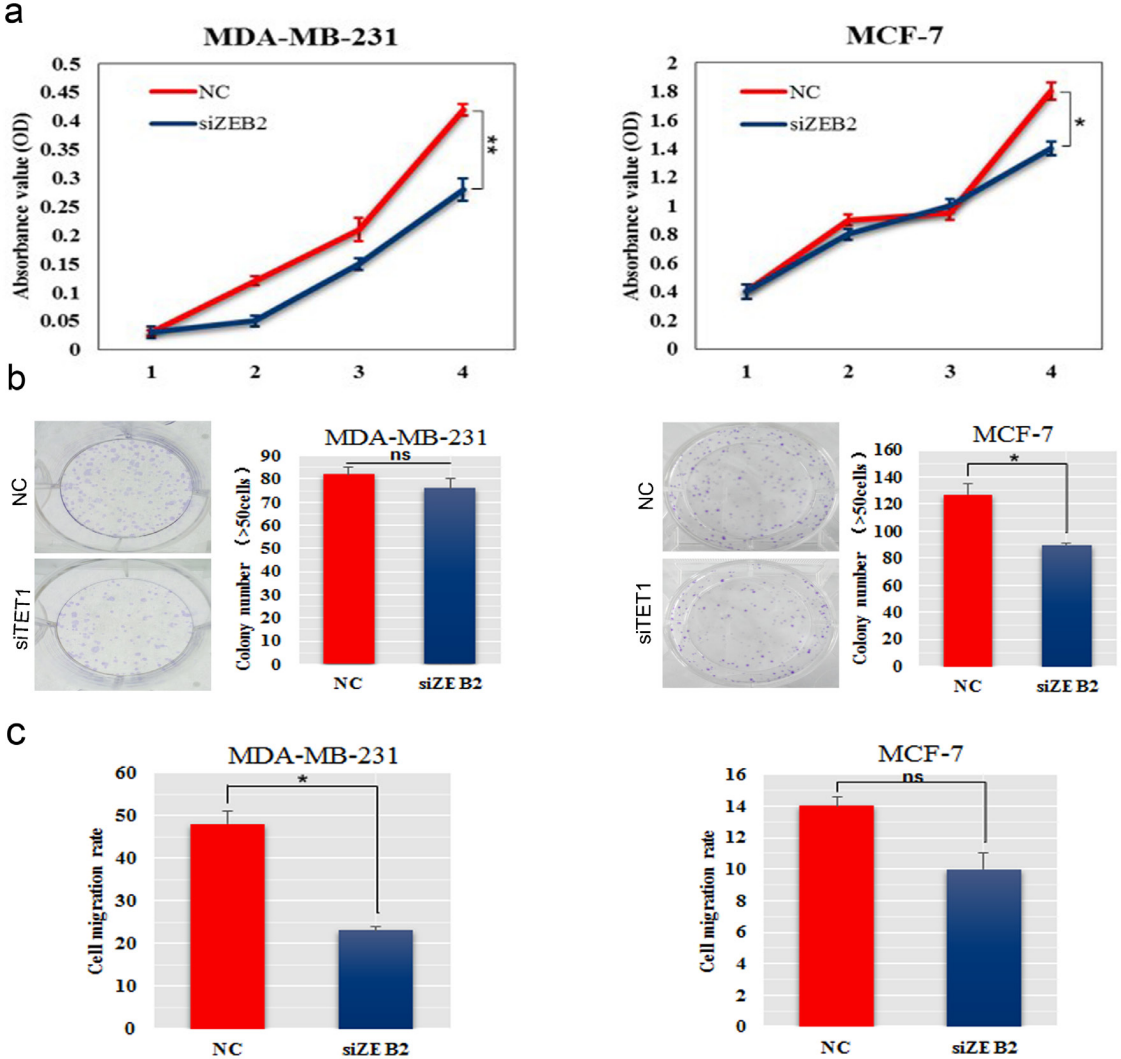
SiZEB2 inhibited BC cells proliferation and metastasis **(a)** The effect of downregulation of ZEB2 on cell proliferation was determined using the CCK-8 assay. **(b)** Colony formation assay was used to detected colony formation ability in MDA-MB-231 and MCF-7 cells transfected with siZEB2. The number of colonies were scored in ten randomly selected fields. Each bar represents the mean of three independent experiments. **(c)** Wound healing assay was to determine invasive ability in MDA-MB-231 and MCF-7 cells transfected with siZEB2.

To determine how ZEB2 affects BC proliferation and metastasis *in vitro*, we inhibited ZEB2 expression in MDA-MB-231 and MCF-7 cells by using small interfering RNA. The CCK-8 assay and colony formation analysis showed that knockdown of ZEB2 significantly decreased cell proliferation and colony formation ability, resprectively, in both BC cell lines (Figure 7a−7b). Invasion assay showed that celluar migration ability was reduced in siZEB2-transfected MDA-MB-231 and MCF-7 cells (Figure 7c).

## DISCUSSION

MiRNAs have important roles in regulating cell cycle and metastasis during cancer development [30]. Efforts have been made to improve cell survival by modulating miRNA levels, which have yielded promising results [31, 32]. In breast cancer, a recent study identified miR-22, which directly targets the TET family, as a promoter of breast cancer metastasis in mouse xenograft models. Moreover, miR-125b, miR-140 and miR-17/20 clusters were reported to inhibit breast cancer progression [33, 34]. Thus, protecting breast cancer cells against metastasis by downregulating pro-proliferation miRNAs or upregulating anti-proliferation miRNAs is a promising strategy. Recently, accumulated evidence has indicated that dysregulation of miR-29b is present in many types of tumors [7, 35]. Low miR-29b expression is positively associated with larger tumor sizes and more advanced cancer stage [36]. In the present study, we found that expression of miR-29b was lower in BC samples than adjacent normal tissue, which suggests that miR-29b could be a biomarker of BC. Though miR-29b was also expressed in adjacent normal tissues and normal breast epithelial cell MCF-10A, it's significantly lower expression in BC cell lines further proved that lower expression of miR-29b could be a indicator of BC. Our observation of low miR-29b expression in breast cancer cells is consistent with a previous study [10]. Furthermore, low expression of miR-29b has been found to have a significant association with poor overall survival in ER-positive and ER-negative breast cancer patients [36]. Our results provide cellular level data that miR-29b is low expressed in the MDA-MB-231 (ER-negative) and MCF-7 (ER-positive) cell lines, which indicates miR-29b is implicated in the migration of malignant breast cells and patient survival.

It is well-known that miR-29b has opposed effects in cancer metastasis according to different cell, cancer types or conditions [37, 38]. In colorectal carcinoma, miR-29b inhibited the proliferation and migration of colorectal cancer cell lines SW480 and HCT116 [35]. In renal cell carcinoma, miR-29b acted as an onco-miRNA by promoting proliferation and invasion ability of SN12-PM6 cells [39]. In cervical cancer, miR-29b could suppress the invasion, EMT procedure and angiogenesis of cervical cancer cells *in vitro*. While treated with cisplatin, chemotherapy-mediated miR-29b expression participates in the initiation and progression of cervical cancer [40]. Chou et al. found that miR-29b inhibited metastasis in breast cancer, and the loss of miR-29b caused a spindle-like morphology and increased mesenchymal marker levels in mouse 4TO7 cells [41]. In our study, we found that miR-29b exerted a tumor-promoting function *in vitro* by promoting proliferation, metastasis, EMT and inhibiting the generation of 5hmC. The discrepancies between our findings and previous reports could be explained, in part, by differences in genetic backgrounds of the cells and methodologies used. Furthermore, we searched for the molecular basis of the miR-29b tumor-promoting function and identified miR-29b-regulated pathways using TargetScan (www.targerscan.org/vert_71), a widely used methodology to identify miRNA targets [42]. Although most of the identified targets are known to be involved in the cancer pathways, many targets have been shown to be essential, but not sufficient individually for both EMT and 5hmC changes. Therefore, we speculate that miR-29b promotes breast cancer cell proliferation and metastasis through regulation of the TET1 gene, which functions and coordinates several biological processes and pathways.

TET1 has been reported to be a biomarker in BC development. The global reduction of 5hmC is a negative prognostic factor for invasive ductal carcinoma, especially for the ER/PR-negative subtype [43, 44]. TET1 participates in DNA demethylation by catalyzing the conversion of 5-methylcytosine to 5-hydroxymethylcytosine [45, 46]. There have been many studies on miR-29b/TET1 being clearly associated with cell development [47]. In one study, Tu et al. found that miR-29b regulation of TET1 contributed to epigenetic regulation during ESC differentiation [48]. In another study, Morita et al. reported that miR-29b directly inhibited TET1 to repress the activity of DNA demethylases [49]. Here, we show that TET1 is a direct target gene of miR-29b, which is supported by the results that overexpression of miR-29b repressed TET1 expression and vice versa. In addition, we further observed that TET1 was down regulated in both miR-29b high- and low- expressing groups, which suggests that low expression of TET1 is a universal marker in miR-29b regulation of BC. Besides, expression of substantial EMT related genes expression were altered after disrutpion of TET1 expression than in miR-29b transfected BC cell lines. This further supports the ideas that miR-29b influence BC metastasis mainly through targeting TET1 and that TET1 plays a crucial role in EMT regulation, which explains the observation that low levels of miR-29b in BC patients promotes breast cancer development.

Epithelial-mesenchymal transition is one of the essential processes involved in the metastasis of breast cancer [50, 51]. E-cadherin, which is a key epithelial cell marker, plays an important role in cancer cell EMT [52]. Indeed, functional loss of E-cadherin in epithelial cells has been considered a hallmark of EMT. In analyzing known genetic EMT regulating factors, we did not find any detectable signal of traditional EMT-related genes, including TWIST1, TWIST2, FOXC1, FOXC2, LBX1 and SIX1 after knockdown of TET1 in MDA-MB-231 cells. However, ZEB2, which has been reported to be negatively correlated with that of the epithelial marker E-cadherin [53], was found to be upregulated in MDA-MB-231 cells transfected with siTET1. A recent study showed that TET1 was also a target gene of miR-29a [54]. However, this report didn't present detailed information on TET1 regulation of EMT- related genes. Although we observed that miR-29b could regulate ZEB2 expression, TargetScan showed that there was no direct connection between miR-29b and ZEB2. However, we demonstrated that the TET1 protein bound directly to the ZEB2 promoter region, as we suggested by ChIP-sequencing (ChIP-seq) data for TET1 in mouse ES cells [55, 56]. Owing to the demethylation activity of TET1, we found that knockdown of TET1 could not only upregulate ZEB2 expression, but also upregulated the methylation level of the ZEB2 promoter, which indicates that TET1 is a negative regulator of the ZEB2 gene both at the transcriptional and translational levels. Therefore, we conclude that miR-29b regulates ZEB2 primarily through a miR-29b/TET1/ZEB2 pathway.

**Figure 8:**
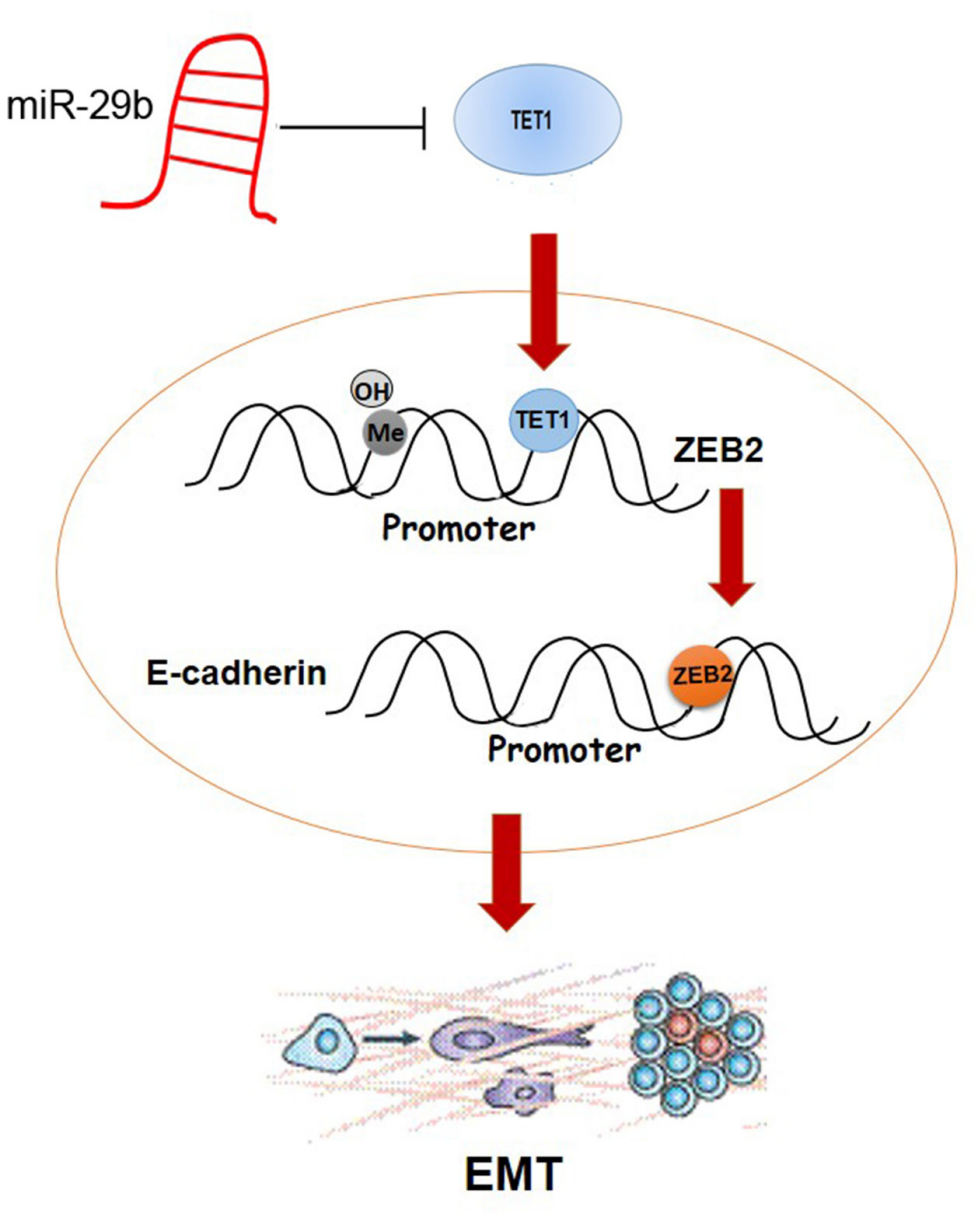
Schematic overview of the molecular function of miR-29b in BC cell lines MiR-29b directly targets TET1 in cytoplasm. After entering into nuleaus, TET1 binds to the promoter region of ZEB2, simultaneously demethylating CpG islands. ZEB2 further binds to E-cadherin promoter, which regulates EMT progress.

In summary, we find that miR-29b affects BC proliferation and metastasis via targeting gene TET1, which regulates EMT-related gene ZEB2 by binding to its promoter and demethylating CpG islands (Figure 8). This study helps to delineate a complex signaling network in breast cancer mediated by miR-29b and presents a miR-29b/TET1/ZEB2 pathway involved in the progression of breast cancer.

## MATERIALS AND METHODS

### Cell culture and transfection

Human breast cancer cell lines (HBL-100, MDA-MB-231 and MCF-7) and the normal human breast epithelial cell line (MCF-10A) were purchased from the Institute of Basic Medical Sciences, Chinese Academy of Medical Sciences (Beijing, China) and cultured in either DMEM or RPMI1640 media (Gibco, USA) supplemented with 10% foetal calf serum (Bioind, Israel), 100 IU/ml penicillin and 100 μg/ml streptomycin (Hyclone, USA). Cells were cultured in a humid environment with 5% CO_2_ at 37 °C.

MiRNAs were transfected at a working concentration of 100 nmol/l using X-trmeGENE siRNA transfection reagent (Roche, Germany). A miR-29b mimic, a nonspecific miR control, anti-miR-29b, a nonspecific anti-miR control, and siRNA against TET1 were all purchased from GenePharma (Shanghai, China). RNA and protein samples were extracted during the exponential phase of growth.

### Tumor tissue samples

All tumor samples and adjacent normal tissue were collected in First Hospital Jilin University. This study was approved by the Ethics Committee of First Hospital Jilin University and all aspects of the study were performed in compliance with the Declaration of Helsinki. The Ethics Committee of the First Hospital Jilin University specifically ensured that informed consent obtained and samples were de-identified so that data were analyzed anonymously.

### QRT-PCR

For qPCR, cDNA was synthesized using a TransScript one-step gDNA Removal and cDNA Synthesis SuperMix (TransGen, China). QPCR was performed using SYBR premix Ex Taq (Takara, Japan), and relative gene expression data were analysed using qRT-PCR and the 2^-ΔΔCT^ method. Primers sequences are listed in Table 1. For miR-29b, qPCR was performed using Hairpin-itTM microRNA and a U6 snRNA Normalization RT-PCR Quantitation Kit (GenePharma, Beijing) on a LightCycler480 (Roche, Germany).

**Table 1:** Primer sequences designed for PCR

Primer	Primer pair sequences (5’ to 3’)
qRT-PCR	
miR-29b	F: CAGACCTGTAGCACCATTTGAA
	R: CACTTCCTCAGCACTTGTTCCTA
U6	F: ATTGGAACGATACAGAGAAGATT
	R: GGAACGCTTCACGAATTTG
SPARC	F:TTGTGGCAAAGAAGTGC
	R:AGAAACCGAAGAGGAGG
C1QTNF6	F:GCCAGGTCCAGCATCACA
	R: CGCTTCTTCGCCTTCTCA
GAPDH	F:GCCTCAAGATCATCAGCAATGCCT
	R: TGTGGTCATGAGTCCTTCCACGAT
TET1	F: ACCTATTCCCCGAATCAAGC
	R: TTGCACGGTCTCAGTGTTACTC
ZEB2	F: CGCTTGACATCACTGAAGGA
	R: CTTGCCACACTCTGTGCATT
Bisulfite-sequencing PCR	
ZEB2	TGTGAATGGTGTGTAT
	ATAAAATTCCACCTCC
Chip-qPCR	
ZEB2	ACTATCTGGATTGAGGACCCG
	TGGCATCATTATCCTCATCACT

QRT-PCR primers were used to detect the relative expression of genes, including miR-29b, U6, SPARC, C1QTNF6, GAPDH, TET1 and ZEB2. Another two ZEB2 primers were specially designed for Bisulfite-sequencing PCR and Chip-qPCR.

### Western blotting

Cells were lysed in RIPA buffer. Protein concentration was measured using a BCA protein assay kit (Thermo Scientific, USA). Equal amounts of cell lysates were subjected to SDS–PAGE, transferred to PVDF membranes, blocked in 5% BSA, incubated with primary antibody overnight and visualized using ECL Detection Reagents (Pierce, USA). Images were acquired using a LAS-4000 Imager (Fuji). Antibodies used include mouse antibodies to *β*-actin (BOSTER #BM0627, 1:200) and rabbit antibodies to E-cadherin (BOSTER #PB0583, 1:200), Vimentin (BOSTER #PB0378, 1:200), and TET1 (Santa Cruz #sc-163443, 1:200) and HRP anti-rabbit and HRP anti-mouse (Bioss #bs-0295G and bs-0296G, 1:5000).

### Immunostaining

Cells were cultured on cover glass in a 24-well plate. To stain the cells, cells were fixed with 4% paraformaldehyde for 20 min and treated with 0.25% Triton X-100 for 15 min. For 5hmc staining, cells were denatured with 2 N HCl and neutralized with 100 mM Tris-HCl (pH 8.5). After blocking in 10% normal blocking serum at room temperature for 1 hour, cells were incubated with rabbit antibodies to E-cadherin (same as above) and Vimentin (same as above) at 4 °C overnight, followed by washing with PBS three times and incubation with a secondary antibody conjugated to Alexa-Fluor-488 (Invitrogen) and Hoechst (Invitrogen) staining for 30 min at 37 °C. Cells were then imaged using a × 10 objective on a Cellomics Cell Insight system. An algorithm measuring the nuclear fluorescence intensity was used for analysis.

### Cell proliferation, invasion and colony formation

Cell proliferation was detected using a Cell Counting Kit-8 (CCK-8). Briefly, 1×10^3^ cells per well were seeded in a 96-well plate, and we measured OD450 on days 0, 1, 2, and 3 after adding CCK-8. For cell invasion, 2×10^5^ cells per well were cultured, and when cells filled the bottom of the well, we scratched a line through the cells and took pictures after 0, 24, and 48 hour(s). For colony formation, cells were cultured in RPMI1640 medium without serum for 12 hours, and then, 5×10^2^ cells per well were seeded in a 6-well plate in RPMI1640 (10% foetal bovine serum) for 2 weeks. Finally, the cells were stained with crystal violet and the colony number was counted.

### Luciferase activity assay

TET1 3’UTR was inserted downstream of the luciferase gene in the PRL-TK vector (Promega, Madison, WI, USA). This vector was designated as wild-type (wt) 3’UTR. Site-directed mutagenesis of the miR-29b binding site in the TET1 3’UTR was carried out using the GeneTailor Site-Directed Mutagenesis System (Invitrogen, USA) and named mutant (mt) 3’UTR. For reporter assays, the wt or mt 3’UTR vector and miR-29b mimic were cotransfected into 293T cells. Luciferase activity was measured 48 h after transfection using the Dual-Luciferase Reporter Assay System (Promega, USA).

### CHIP assay

Cells were fixed and cross-linked with 1% formaldehyde. Cross-linked chromatin was sheared by using a sonicator. The antibody used for immunoprecipitation was anti-TET1 (Santa Cruz Biotechnologies, USA). Precipitated DNA was purified and then analyzed by qPCR with primer specific for the ZEB2 region. Primers used are listed in Table 1.

### Statistical analysis

All experiments were replicated at least three times. Statistical analysis was performed using SPSS (Statistical Package for the Social Sciences) 19.0 software (Chicago, IL, USA) by one-way ANOVA. Differences were considered statistically significant when *P* < 0.05, and *P* < 0.01 was considered extremely significant.

## SUPPLEMENTARY MATERIALS FIGURE



## Abbreviations

BC: Breast cancer
C1QTNF6: C1q and tumor necrosis factor related protein 6
DNMT: DNA methyltransferases
EMT: Epithelial–mesenchymal transition
ER: Estrogen receptor
miRNAs: MicroRNAs
NC: Negative control
PGCs: Primordial germ cells
QRT-PCR: Quantitative real-time polymerase chain reaction
SPARC: Secreted protein acidic and cysteine rich
TDG: Thymine DNA glycosylase
TET: Ten-eleven translocation
UTR: Untranslated region
ZEB2: Zinc finger E-box-binding homeobox
5hmC: 5-hydroxymethylcytosine
