# Supplementary material for: MiR-29b/TET1/ZEB2 signaling axis regulates metastatic properties and epithelial-mesenchymal transition in breast cancer cells
Source: Oncotarget. 2017 Oct 31;8(60):102119–33. doi: 10.18632/oncotarget.22183 (PMC5731940; doi:10.18632/oncotarget.22183)
Supplement: Supplementary file 1 [file oncotarget-08-102119-s001.pdf]

MiR-29b/TET1/ZEB2 signaling axis regulates metastatic properties and epithelial-mesenchymal transition in breast cancer cells

SUPPLEMENTARY MATERIALS

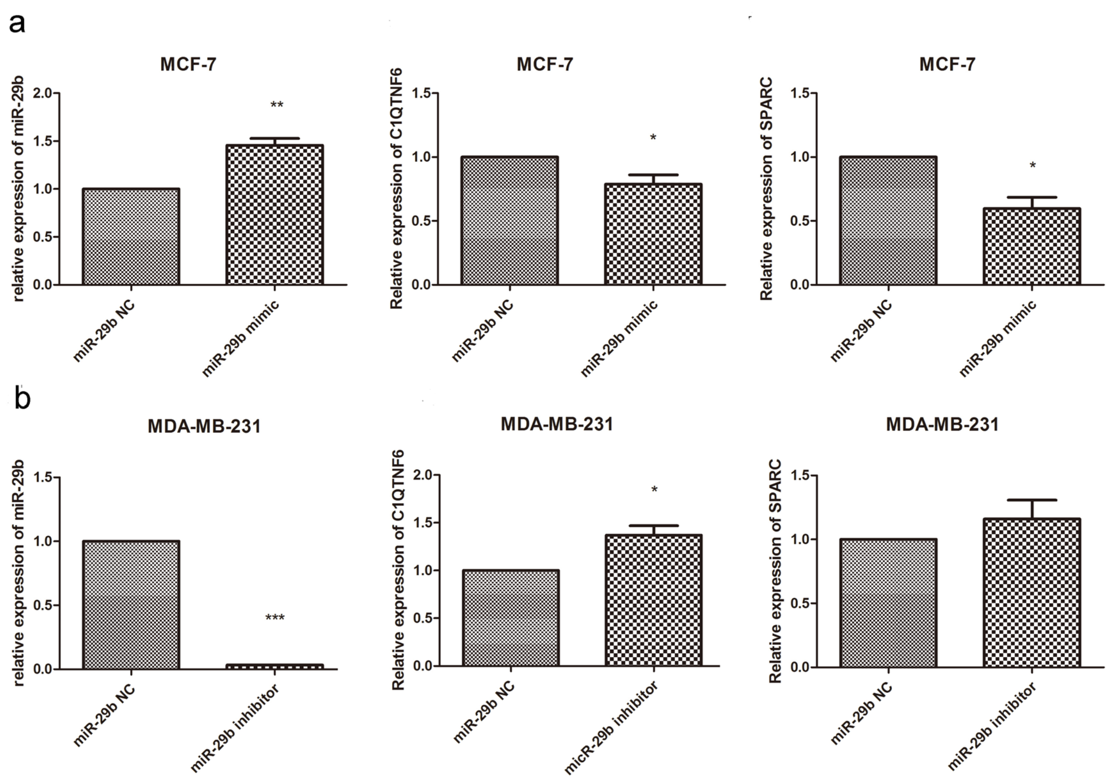

Supplementary Figure 1: The expression of miR-29b, C1QTNF and SPARC in miR-29b mimic or inhibitor transfected BC cell lines. **(a)** The relative expression of miR-29b, C1QTNF and SPARC was detected in miR-29b mimic transfected MDA-MB-231 cells. **(b)** MiR-29b, C1QTNF and SPARC expression level in miR-29b inhibitor transfected MCF-7 cells. All data are expressed as the mean ± S.E.M. Asterisks denote significant effects; $*P < 0.05$; $**P < 0.01$.
